# Patterns of HER2 Expression in Metastatic Prostate and Urothelial Cancers: Implications for HER2-Targeted Therapies

**DOI:** 10.1158/2767-9764.CRC-25-0069

**Published:** 2025-08-25

**Authors:** Hyun Jung Lee, Roman Gulati, Erolcan Sayar, Radhika A. Patel, Pushpa Itagi, Helen M. Richards, Thomas Persse, Sonali Arora, Ilsa Coleman, Mohamed Adil, Samantha L. Schuster, Farinaz Shokri, Jonathan L. Wright, Evan Y. Yu, Lawrence D. True, Meagan Chambers, Jessica E. Hawley, Heather H. Cheng, Michael T. Schweizer, Petros Grivas, Peter S. Nelson, R. Bruce Montgomery, Andrew C. Hsieh, Funda Vakar-Lopez, Colm Morrissey, Hung-Ming Lam, Gavin Ha, Maria S. Tretiakova, Michael C. Haffner, Ruben Raychaudhuri

**Affiliations:** 1 Department of Laboratory Medicine and Pathology, University of Washington, Seattle, Washington.; 2 Department of Pathology, Pusan National University School of Medicine, Yangsan, Korea.; 3 Pusan National University Yangsan Hospital, Yangsan, Korea.; 4 Clinical Research Division, Fred Hutchinson Cancer Center, Seattle, Washington.; 5 Division of Human Biology, Fred Hutchinson Cancer Center, Seattle, Washington.; 6 Department of Urology, University of Washington, Seattle, Washington.; 7 Division of Hematology and Oncology, Department of Medicine, University of Washington, Seattle, Washington.; 8 Department of Pathology, Stanford University, Stanford, California.

## Abstract

HER2 is an oncogenic driver in multiple cancers and a predictive biomarker for HER2-targeted therapies. Although HER2-directed therapies like fam-trastuzumab deruxtecan are approved for HER2-positive breast and other solid tumors, the landscape of HER2 expression in advanced prostate cancer and urothelial carcinoma remains inadequately characterized. We evaluated HER2 protein expression in metastatic prostate cancer and urothelial carcinoma using a validated IHC assay on tissue microarrays constructed from the University of Washington Tissue Acquisition Necropsy Program. HER2 expression was scored using standardized gastric cancer criteria. Genomic analysis of *ERBB2* alterations was conducted on a subset of samples. HER2 expression heterogeneity and its relationship with other surface markers were also evaluated. In the prostate cancer cohort (*n* = 52 patients, 1–19 tumors per patient), HER2 expression was low, with no 3+ expression observed and only five (10%) patients exhibiting 2+ expression. Low-level *ERBB2* copy-number gains were observed in some tumors but did not correlate with HER2 protein expression (*P* = 0.2). In urothelial carcinoma (*n* = 20, 2–6 tumors per patient), HER2 expression was more frequent, with ≥2+ expression observed in six (30%) cases and 3+ expression observed in three (15%) cases in at least one tumor. Urothelial carcinoma samples showed less heterogeneity, with more consistent expression across metastases. HER2 overexpression is rare and heterogeneous in metastatic prostate cancer, limiting its utility as a therapeutic target. HER2 expression is more prevalent and uniform in urothelial carcinoma. These findings underscore the importance of comprehensive HER2 assessment in advanced urothelial carcinoma and suggest that success of HER2-directed therapies in prostate cancer will require careful case selection.

**Significance:**

This study demonstrates that HER2 is rarely overexpressed in metastatic prostate cancer but is more common and consistent in urothelial carcinoma. These findings highlight the need for HER2 testing in urothelial cancer and suggest that HER2-targeted therapies in prostate cancer will require careful patient selection.

## Introduction

HER2 is a cell surface protein and member of the EGFR family encoded by the gene *ERRB2* (1). Activation of HER2 promotes downstream mitogenic signaling pathways (2) and overexpression of HER2 can lead to unchecked cellular proliferation and tumorigenesis (3, 4).

HER2 is best known as a key oncogenic driver of breast cancer, in which it is found to be overexpressed in about 20% of cases, either because of *ERBB2* amplification or activating somatic mutations (5). HER2 overexpression, as determined by IHC or by the demonstration of *ERRB2* gene amplification by FISH, is a robust predictive biomarker for HER2-targeted therapies such as trastuzumab and pertuzumab (6–9).

Fam-trastuzumab deruxtecan (T-DXd) is an antibody–drug conjugate (ADC) consisting of the anti-HER2 monoclonal antibody trastuzumab coupled to the cytotoxic topoisomerase I inhibitor deruxtecan via a cleavable linker (10, 11). In contrast to other HER2-directed therapies, T-DXd shows efficacy in patients with breast cancer with low HER2-expressing tumors, defined as either 1+ by IHC or 2+ without gene amplification by FISH (12). T-DXd was recently also evaluated in a pan-cancer cohort leading to the tumor-agnostic approval of this ADC for all patients with solid tumors that show high (3+) HER2 expression on standardized IHC assessment (13, 14). Notably, although the current approval requires 3+ HER2 expression, T-DXd also showed activity in cases with 2+ expression (13).

With this recent approval, there is renewed interest in defining the landscape of HER2 expression in advanced prostate and urothelial cancers. Prior studies assessed HER2 expression in prostate cancer predominantly in localized disease (15–17). Additional retrospective studies suggested that HER2 expression may increase in castration-resistant disease, suggesting that the rate of HER2 overexpression in advanced prostate cancer may be underestimated (16, 18, 19). Furthermore, the molecular determinants of HER2 expression in prostate cancer are largely unexplored. Therefore, there is a significant knowledge gap in the field’s understanding of HER2 in prostate cancer.

Compared with the relatively limited data on HER2 expression in prostate cancer, the patterns of expression in urothelial carcinoma are better characterized (20, 21). However, there is a clear need to further define the expression of HER2 in advanced urothelial carcinoma, assess the heterogeneity across different metastatic sites, and determine the co-expression pattern with other relevant ADC targets, such as nectin cell adhesion molecule 4 (NECTIN-4) and trophoblast cell surface antigen 2 (TROP2).

To address these clinically relevant questions, we evaluated the expression landscape of HER2 in metastatic prostate cancer and urothelial carcinoma using a validated HER2 IHC assay and determined genomic features underlying differential expression. We leveraged the well-annotated University of Washington rapid autopsy cohort, which allowed for a comprehensive analysis of metastatic and primary tumor tissues and a detailed assessment of patterns of inter- and intra-patient expression heterogeneity. Collectively, these data provide relevant insights into the potential use and further clinical development of T-DXd or other HER2-directed therapeutics in these malignancies.

## Materials and Methods

### Study approval

This study was approved by the Institutional Review Board of the University of Washington (protocol no. 2341) and complied with the ethical regulations including the Declaration of Helsinki. Written informed consent was obtained from all participants in this study.

### Tissue samples

Tissue samples were collected from patients who succumbed to metastatic prostate cancer or urothelial carcinoma and who participated in the University of Washington Tissue Acquisition Necropsy Autopsy Program (22, 23). Under this protocol, approved by the Institutional Review Board of the University of Washington, all patients provided written informed consent for a rapid research autopsy and tissue procurement. Procedures were carried out with a mean post-mortem interval of 4.9 hours (range, 2–23 hours) for the prostate cancer and 4.4 hours (range, 3–12 hours) for the urothelial carcinoma cohort. Pathologic features of the cohorts are described in Supplementary Tables S1 and S2. Following a standard protocol, primary tissue (in patients who had not previously undergone surgery), all grossly identifiable soft tissue, and visceral metastases were sampled, and systematic biopsies were obtained to assess the vertebral and non-vertebral bones. Metastases were divided for both formalin-fixed, paraffin-embedded processing and flash freezing. Formalin-fixed, paraffin-embedded tissue blocks were used for IHC-based studies, whereas genomics and transcriptomics studies were largely conducted on frozen tissues. All bone specimens underwent decalcification with 10% formic acid before paraffin embedding. Tissue microarrays with a core diameter of 1 mm were constructed as described previously (24). Each metastatic site (i.e., tumor) was sampled with at least two cores to improve tumor representation.

### IHC staining

For all immunostaining experiments, 5-μm-thin tissue microarrays were stained using the PATHWAY anti-HER2/neu rabbit monoclonal antibody 4B5 (Ventana/Roche Tissue Diagnostics). Immunoreactivity was scored by two pathologists (M.S. Tretiakova and H.J. Lee) independently by applying the gastric cancer HER2 scoring criteria as established in the ToGA trial (25, 26). Unlike the criteria for breast carcinoma, the gastric cancer criteria do not require complete membranous staining for positivity; however, luminal-only staining is considered negative. Cytoplasmic HER2 in the absence of membranous staining is also considered negative. Cases with discrepant staining scores were reviewed by two independent pathologists (E. Sayar and M.C. Haffner) to reach consensus. Additional IHC data for TROP2, NECTIN-4, and prostate-specific membrane antigen (PSMA) were scored by multiplying the staining level (0–2, with higher number representing more intense immunoreactivity) with the percentage of cells at each level, resulting in a total H-score. The phenotype of prostate cancer tumors was determined by the relative expression of AR, NKX3.1, SYP, and INSM1 as previously described (24, 27). These secondary IHC results were published previously for the prostate cancer cohort (24, 28, 29).

### Genomic and transcriptomic analysis

For genomic analysis, whole-exome sequencing datasets were analyzed (24). Single-nucleotide variant (SNV) calling was performed using MuTect 2 (GATK version 4.1.8.1), Strelka 2 (version 2.9.2), and VarScan 2 (version 2.4.4; refs. 30–32). Insertions and deletions were called using SvABA (33). All SNV and insertion and deletion calls were annotated using ANNOVAR (release 20200607; ref. 34). TitanCNA version 1.23.1 was used for copy number calling (35). Gene-level copy number calls were derived from TitanCNA’s segments using GenomicRanges version 1.38.0. Gene-level copy number calls were converted to ploidy-adjusted copy number (PACN) using TitanCNA’s estimated sample ploidy. The following thresholds were used to define copy number events: amplification: PACN >2.5, gain: PACN ≥1.5, deletion: PACN ≤0.5, and homozygous deletion: PACN = 0. As PACN may obscure focal amplifications, the relationship between unadjusted *ERBB2* copy number was also studied, in which copy-number gain was defined as three to five total copies and amplification >5.

RNA sequencing (RNA-seq) data of bulk flash-frozen tissues from University of Washington Tissue Acquisition Necropsy cohorts were processed as described previously (24, 28). All subsequent analyses were performed using R. Gene abundance was quantified using GenomicAlignments and limma (36, 37). Molecular subtype classification (androgen receptor/neuroendocrine status) was performed as described previously (27).

### Statistical analysis

Probabilities of HER2 expression scores conditional on index samples were estimated by relative proportions of samples with each HER2 expression score among patients having at least one sample with the index score. Associations between HER2 expression and H-scores of relevant cell surface antigens (TROP2 and NECTIN-4 or PSMA) were evaluated using linear regressions with random intercepts to account for repeated measurements from the same patients; for prostate cancer, these associations were stratified by phenotype. Associations between genomic alterations of the *ERBB2* gene locus and HER2 expression were evaluated using two-sided Fisher exact tests; for urothelial carcinoma, deletions were omitted.

### Data availability

All results associated with this study are present in the article or supplementary materials. Transcriptomic and genomic data used in this study pertaining to prostate cancer have been published (Gene Expression Omnibus: GSE205056, GSE126078, and GSE147250). Transcriptomic data from the urothelial carcinoma cohort have been published with this article (GSE300195), and a subset of the raw sequencing data was previously submitted to the Database of Genotypes and Phenotypes (accession phs001797.v1.p1; ref. 23). All other materials and data used in the analyses are available upon reasonable request.

## Results

### Prostate cancer cohort

**Figure 1:**
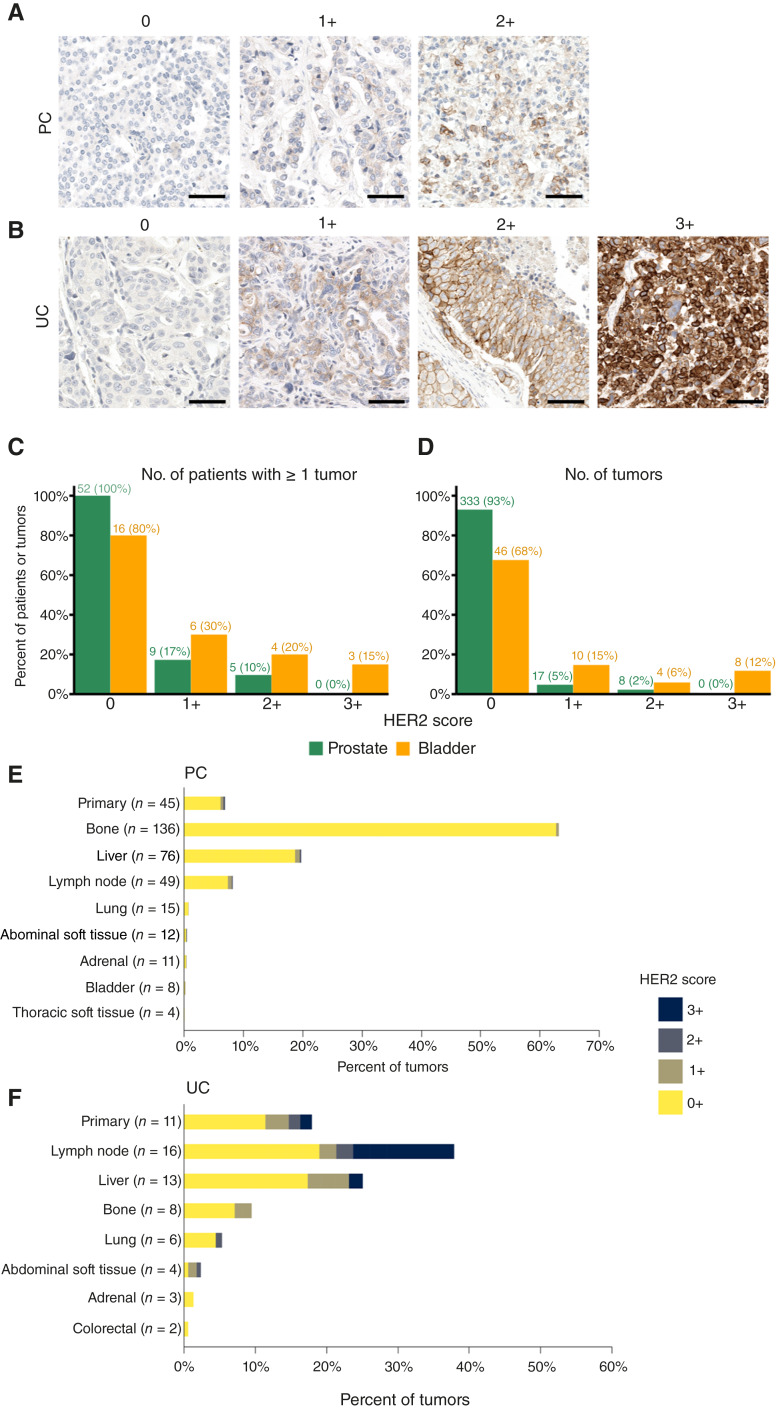
**A** and **B,** Representative micrographs of HER2 IHC staining in prostate cancer (**A**) and urothelial cancer (**B**) autopsy specimens. **C,** Number of patients with at least one tumor with the indicated HER2 IHC score, color coded by cancer type. **D,** Number of tumors across all patients with the indicated HER2 IHC score, color coded by cancer type. **E** and **F,** Percent of tumors expressing the indicated level of HER2 staining by IHC according to the tumor site sampled in prostate (**E**) and urothelial (**F**) cancer cohorts. Anatomic sites with only one representative tumor were excluded. PC, prostate cancer; UC, urothelial carcinoma.

We evaluated HER2 expression in 358 tumor samples from 52 patients (median seven samples per patient; range, 1–19). The median age at diagnosis was 60.6 years. At the time of diagnosis, 46 patients had a Gleason score available, of whom 27 (58.7%) were at the grade group of 5. Of the 52 patients, 51 (98%) cases received androgen deprivation therapy (one patient had *de novo* neuroendocrine carcinoma and did not receive hormonal therapy) and had developed castration-resistant disease at the time of death, and the median survival from cancer diagnosis was 5.2. We did not observe any tumors with 3+ HER2 expression (Fig. 1A and C); five patients exhibited HER2 2+ expression in at least one tumor.

Among patients with HER2 2+ expression at one site, the likelihood that a second tumor from the same patient was 2+ was 10% (Table 1). Most cases with an index tumor sample demonstrating HER2 2+ expression exhibited either HER2 0+ (74%) or 1+ expression (16%). HER2 expression did not seem to vary significantly by anatomic site (Fig. 1E).

**Table 1:** Estimated probabilities of HER2 expression conditional on an index sample

Cohort	HER2 expression in index tumor sample	Probability of HER2 expression in other samples			
		0+	1+	2+	3+
Prostate	0+	0.92	0.05	0.02	0.00
​	1+	0.80	0.12	0.08	0.00
​	2+	0.74	0.16	0.10	0.00
Bladder	0+	0.87	0.11	0.02	0.00
​	1+	0.47	0.40	0.13	0.00
​	2+	0.33	0.44	0.00	0.22
​	3+	0.00	0.00	0.12	0.88

Note: Probabilities of HER2 expression in other samples may not sum to 1 because of rounding.

**Figure 2:**
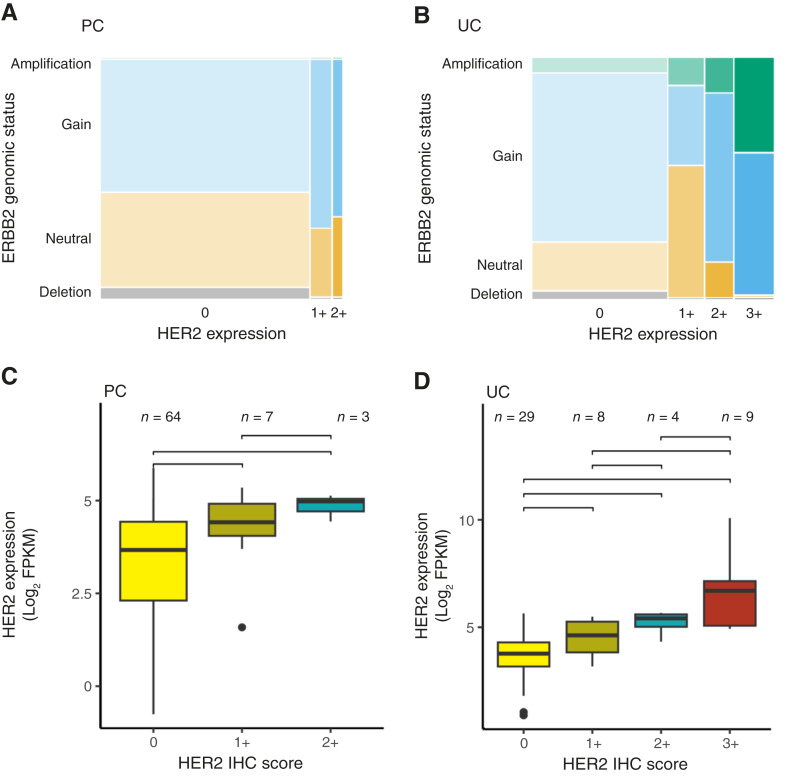
**A** and **B,** Mosaic plots indicating the relationship between ERBB2 genomic status as assessed by unadjusted copy number and HER2 IHC in (**A**) prostate and (**B**) urothelial cancers. **C** and **D,** Box plots indicating the relationship between HER2 RNA expression with (**C**) prostate and (**D**) urothelial cancers. PC, prostate cancer; UC, urothelial carcinoma.

We further analyzed the relationship between genomic alterations of the *ERBB2* gene locus with HER2 expression by IHC. From 44 patients, 85 tumor samples had both sequencing and IHC performed. Sequencing of prostate cancer samples was of high quality, with a median mean coverage of 31.8× (range, 16–43×) and a median percentage of bases covered at 30× of 44.4%. One patient had an SNV of unknown significance (p.R190Q) in the *ERBB2* gene; the sequenced site (a lymph node) was associated with HER2 2+ expression. No amplifications (PACN ≥2.5) were observed; however, low-level copy-number gains were observed in 14 tumors from seven cases. No association between ERBB2 PACN and HER2 expression by IHC was observed (*P* = 0.2; Supplementary Fig. S1A). Unadjusted *ERBB2* copy number similarly did not correlate with HER2 expression (*P* = 0.9; Fig. 2A). From 42 patients, 74 tumors had both RNA-seq and an assessment of HER2 expression by IHC. Although there was a trend toward higher *ERBB2* transcription in tumors with higher HER2 expression by IHC, the association was not statistically significant (Fig. 2C).

**Figure 3:**
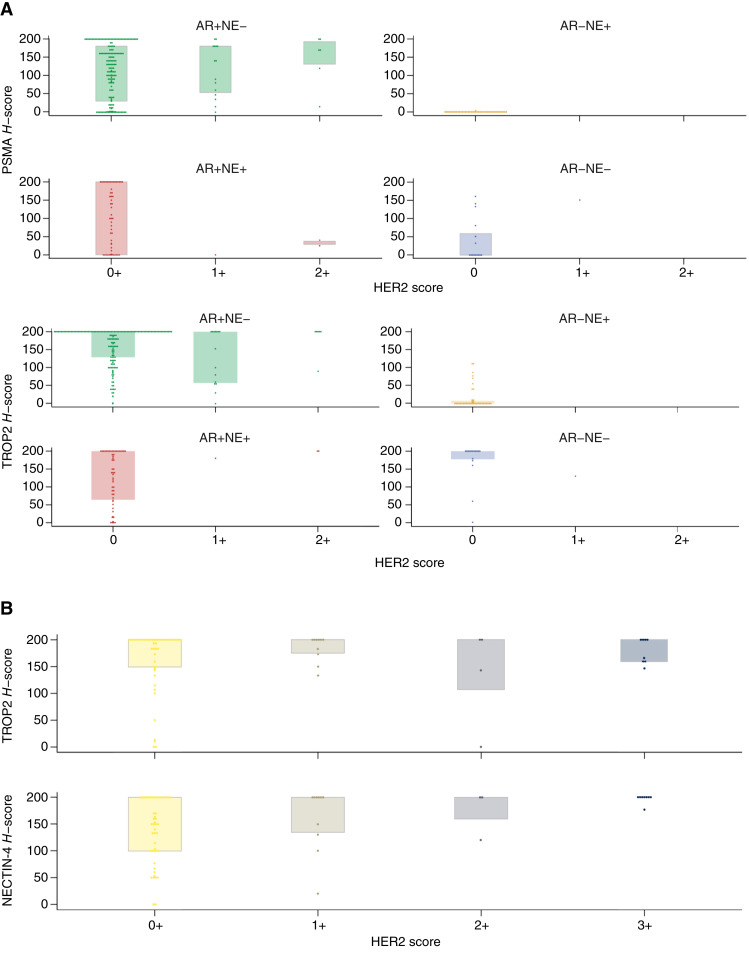
**A** and **B,** Box plots indicating the relationship between HER2 score with protein expression of key cell surface antigens as measured by IHC in (**A**) prostate cancer and (**B**) urothelial cancer.

Next, we determined how HER2 expression varied with the expression of other key cell surface antigens (PSMA and TROP2), stratified by prostate cancer phenotype (Fig. 3A). Relationships between HER2 expression with other antigens were not distinguishable from null associations (i.e., data were consistent with zero-slope regression lines) in this exploratory analysis. Despite relatively low overall protein expression of HER2, especially as compared with PSMA and TROP2, these surface antigens were expressed at a similar level at the RNA transcriptomic level (Supplementary Fig. S2A).

### Urothelial carcinoma cohort

HER2 expression by IHC was quantified in 68 tumors from 20 patients (median three sites per case; range, 2–6). Fifteen cases were bladder in origin and five were upper tract. Patients with variant bladder cancer histologies were included. The median overall survival from metastatic disease in this cohort was 1.5 years. Of the 20 patients, 13 (65%) received platinum-based chemotherapy and 12 of 20 (60%) patients received anti-PDL1/-PD1 therapy. HER2 3+ expression was observed in tumors from three (15%) patients (Fig. 1B and D). Among patients with at least one HER2 3+ tumor, the likelihood of a second tumor exhibiting HER2 2+ or 3+ was 12% or 88%, respectively (Table 1). Among patients with at least one HER2 ≥2+ tumor, the likelihood of a second tumor exhibiting HER2 ≥2+ was 22%. Tumor samples obtained from lymph node metastases (44%) had numerically higher proportion of HER2 ≥2+ compared with those from primary tissue (18%; Fig. 1E).

*ERBB2* genomic status was assessed in 59 tumors from 20 patients. Whole-genome sequencing of bladder cancer samples demonstrated high-quality coverage, with a median mean coverage of 78× (range, 75–94×) and a median of 92% of bases covered at ≥30 depth. *ERBB2* coding mutations were observed in three patients across four metastatic sites; however, only one patient was found to have a known pathogenic activating mutation. In one patient, multiple *ERRB2* mutations, including the activating p.S310F alteration, were detected in the primary tumor, which exhibited HER2 2+ expression. No mutations were observed in other metastatic sites from this patient (liver and lung), which were also negative (0) for HER2 expression.

A second patient had multiple nonsense variants detected in a lung lesion. HER2 expression was 0 in this lesion. These variants were not detected in other sites of disease (lymph node, liver, and bladder primary) in this patient. A third patient had multiple *ERBB2* SNVs of unknown significance detected in lymph nodes but not in the primary tumor. This patient was negative for HER2 expression in all metastatic sites.

*ERBB2* PACN gain was seen in seven tumors from four cases, and the association between *ERBB2* genomic status and HER2 protein expression was not statistically significant (*P* = 0.06; Supplementary Fig. S1B). Only one patient had *ERBB2* PACN amplification, which was associated with HER2 3+ expression. However, a statistical association was observed between unadjusted copy number with HER2 expression (*P* = 0.028; Fig. 2B).

RNA-seq was performed on 50 tumors from 20 cases. We found an association between increasing *ERBB2* RNA expression with higher HER2 expression by IHC (Fig. 2D). We evaluated associations between HER2 expression and cell surface antigens TROP2 and NECTIN-4 (Fig. 3B). We estimated that each one-point increase in the HER2 score was associated with a 19-point increase in NECTIN-4 H-score (95% confidence interval, 4–35), but the relationship with TROP2 was not distinguishable from a null association.

## Discussion

In this study, we evaluated HER2 protein expression patterns in metastatic prostate cancer and urothelial carcinoma leveraging tissues procured as part of the University of Washington Tissue Necropsy Program. Our analyses revealed substantial differences in HER2 expression between prostate cancer and urothelial carcinoma.

We found that HER2 expression in prostate cancer is generally low, with no cases of 3+ expression observed across 358 tumors from 52 cases. Only a small number of cases exhibited HER2 2+ expression; however, even among these cases, relatively few other metastatic sites exhibited HER2 ≥2+ expression. This finding suggests that, even if one metastatic prostate cancer lesion exhibits HER2 ≥2+ staining, it is probable that other lesions may not exhibit the same degree of expression. This observation underscores the potential challenges in utilizing HER2-targeted therapies in prostate cancer, as homogeneous expression across metastatic sites may be relevant to the therapeutic activity of agents such as T-DXd. Although ADCs are known to exert a bystander effect, in which the cytotoxic payload can affect neighboring HER2-negative cells, the degree of this effect can vary and may not fully compensate for marked intra-tumoral heterogeneity or be relevant for marked inter-tumoral heterogeneity, particularly if entire lesions lack HER2 expression altogether. Our findings suggest the need to proceed with caution with interpreting HER2 expression from a single metastatic biopsy in patients with prostate cancer. Furthermore, although HER2 expression has been associated with neuroendocrine differentiation and androgen-indifferent biology, the high-grade neuroendocrine prostate cancer cases in our cohort uniformly did not express HER2 (medRxiv 11:2024:02.09.24302395; refs. 38, 39). Intriguingly, the RNA expression of *ERBB2* was observed at levels similar to the highly expressed TROP2 and PSMA, suggesting that posttranscriptional factors may have an influence on the low level of HER2 expression observed in prostate cancer.

This study is the largest assessment of HER2 expression in metastatic prostate cancer. Previous reports on HER2 expression primarily focused on localized disease, with conflicting results and expression frequencies ranging from 12% to 23% (15, 17, 40). Some of this variability may stem from differences in the assay [e.g., use of the Ventana PATHWAY anti-HER2 (4B5) vs. Dako HercepTest PharmDx (GE001)] used in these studies as well as the scoring algorithm which was used. It is also possible that HER2 expression is higher in localized, hormone-sensitive disease compared with the castration-resistant, advanced metastatic cases examined here. However, most studies have found a relative increase in expression in patients with more advanced, aggressive disease (19, 41, 42). From a pragmatic standpoint, the patient population included in this study is likely the most relevant for potential T-DXd use, which, for prostate cancer, is currently only approved for those without other satisfactory treatment options (13).

Based on our evaluation, metastatic prostate cancer exhibits low HER2 expression overall. Even when individual tumors show reactivity, the high inter-lesional heterogeneity in expression suggests that HER2-directed therapies should primarily be considered in metastatic prostate cancer cases in which HER2 expression has been confirmed across more than one site (38).

The findings in prostate cancer contrast to our observations in urothelial carcinoma, in which HER2 expression was present more frequently, with HER2 ≥2+ and 3+ expressions observed in 20% and 15% of patients, respectively. This expression frequency is on par with prior studies, which range from 20% to 50% (20, 43–46). These results underscore the importance of obtaining HER2 staining on all patients with metastatic urothelial carcinoma given the potential for the use of T-DXd and other HER2-targeting agents.

One patient in the urothelial carcinoma cohort (5%) was found to have an activating mutation in *ERBB2*. This is in line with prior series documenting a mutation frequency of about 5% to 10% in patients with metastatic urothelial carcinoma (47–49). We also observed a weak association between HER2 and NECTIN-4 protein expression, which has previously been observed and attributed to underlying shared luminal phenotype (50, 51). No strong association between TROP2 and HER2 expression was noted, which is likely because of more uniform expression of TROP2 across urothelial carcinoma phenotypes.

Additionally, we found a higher degree of concordance in the urothelial carcinoma cohort as compared with the prostate cancer cohort with respect to HER2 expression between metastatic sites, especially in patients with HER2 3+ disease. Consistent with prior observations, we found a higher prevalence of HER2 overexpression in lymph node tissue compared with primary tumor tissue (44, 52). This suggests that obtaining metastatic tissue for assessment of HER2 status rather than utilizing primary tissue may potentially increase the number of patients eligible for HER2-targeted therapies. Although HER2 expression was generally higher in metastatic sites, we note that the one activating *ERBB2* mutation present in our cohort was detected in the primary site, which exhibited HER2 2+ expression, whereas other sites of disease had no HER2 reactivity.

In prior studies, *ERBB2* gene amplification generally correlated with higher expression on IHC, a relationship that has been observed with other key cell surface markers such as NECTIN-4 (53–56). In the urothelial carcinoma cohort, we observed a strong correlation between unadjusted copy number HER2 expression. The association was weaker with PACN. Importantly, a number of tumors with HER2 2+ and 3+ did not exhibit amplification, highlighting the importance of IHC testing even if amplification is not observed on next-generation sequencing.

This study has several limitations. Patients enrolled in the Tissue Acquisition Necropsy Program at our institution may not be reflective of the general population. This population is heavily pretreated, which may potentially influence HER2 expression. Furthermore, our assessment of HER2 status is cross-sectional rather than longitudinal, and we may have missed changes in expression over time. As HER2 IHC testing and sequencing analysis were conducted on adjacent but not identical tissue specimens, intratumoral spatial heterogeneity may therefore also potentially have limited the correlation between genomic/transcriptomic findings and protein expression. Additionally, the sample size for genomic analysis was small; therefore, it is challenging to make robust conclusions with regard to the relationship between copy-number variations, genomic mutations, and protein expression. Differences in sites sampled between the prostate cancer and urothelial carcinoma cohorts may also have influenced our assessment of inter-patient heterogeneity, and the higher degree of concordance in the urothelial carcinoma cohort as compared with the prostate cancer cohort therefore requires further validation.

Lastly, as with all tissue-based analyses, pre-analytic variables could have influenced the results. However, it is important to note that the tissue procurement, processing, and staining protocols for prostate cancer and urothelial carcinoma rapid autopsy samples were the same (23, 24). Therefore, pre-analytic factors are unlikely to account for the significant differences in HER2 expression observed between prostate cancer and urothelial carcinoma.

In summary, our study highlights significant differences in HER2 expression patterns between metastatic prostate cancer and urothelial carcinoma, with low and heterogeneous expression in prostate cancer limiting the general applicability of HER2-targeted therapies, whereas the higher and more consistent expression in urothelial carcinoma supports its potential role as a therapeutic target although still in selected cases.

## Supplementary Material

Supplementary Table 1 Prostate cancer cohort supplemental table

Supplementary Table 2 UC cohort supplemental table

Figure S1 Supplemental figure 1

Figure S2 Supplemental figure 2
