# Supplementary figures and images for: Patterns of HER2 Expression in Metastatic Prostate and Urothelial Cancers: Implications for HER2-Targeted Therapies
Source: Cancer Res Commun. 2025 Aug 25;5(8):1419–28. doi: 10.1158/2767-9764.CRC-25-0069 (PMC12375912; doi:10.1158/2767-9764.CRC-25-0069)

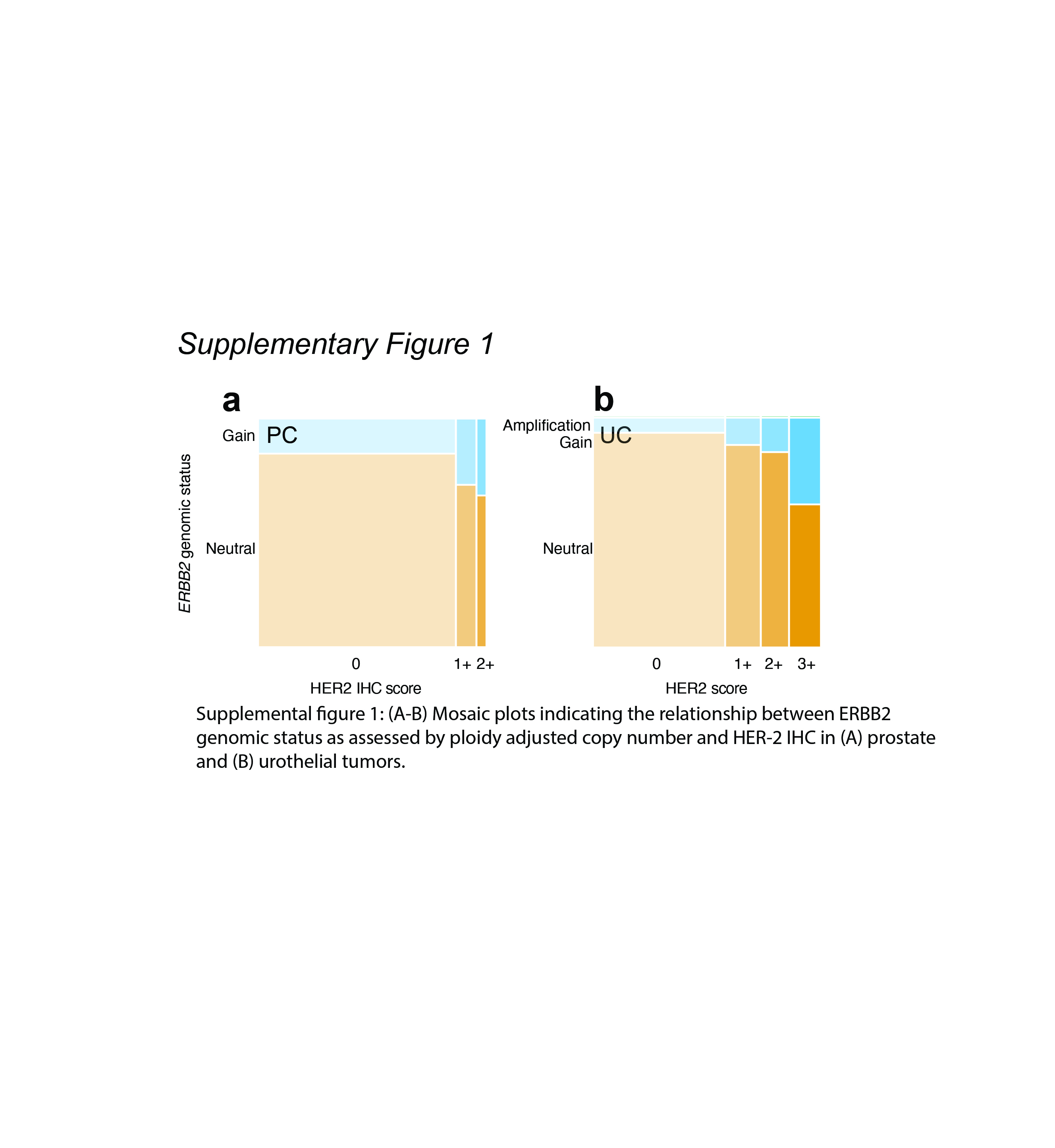

Supplement: Figure S1 — Supplemental figure 1 [file crc-25-0069_figure_s1_suppsf1.jpeg]

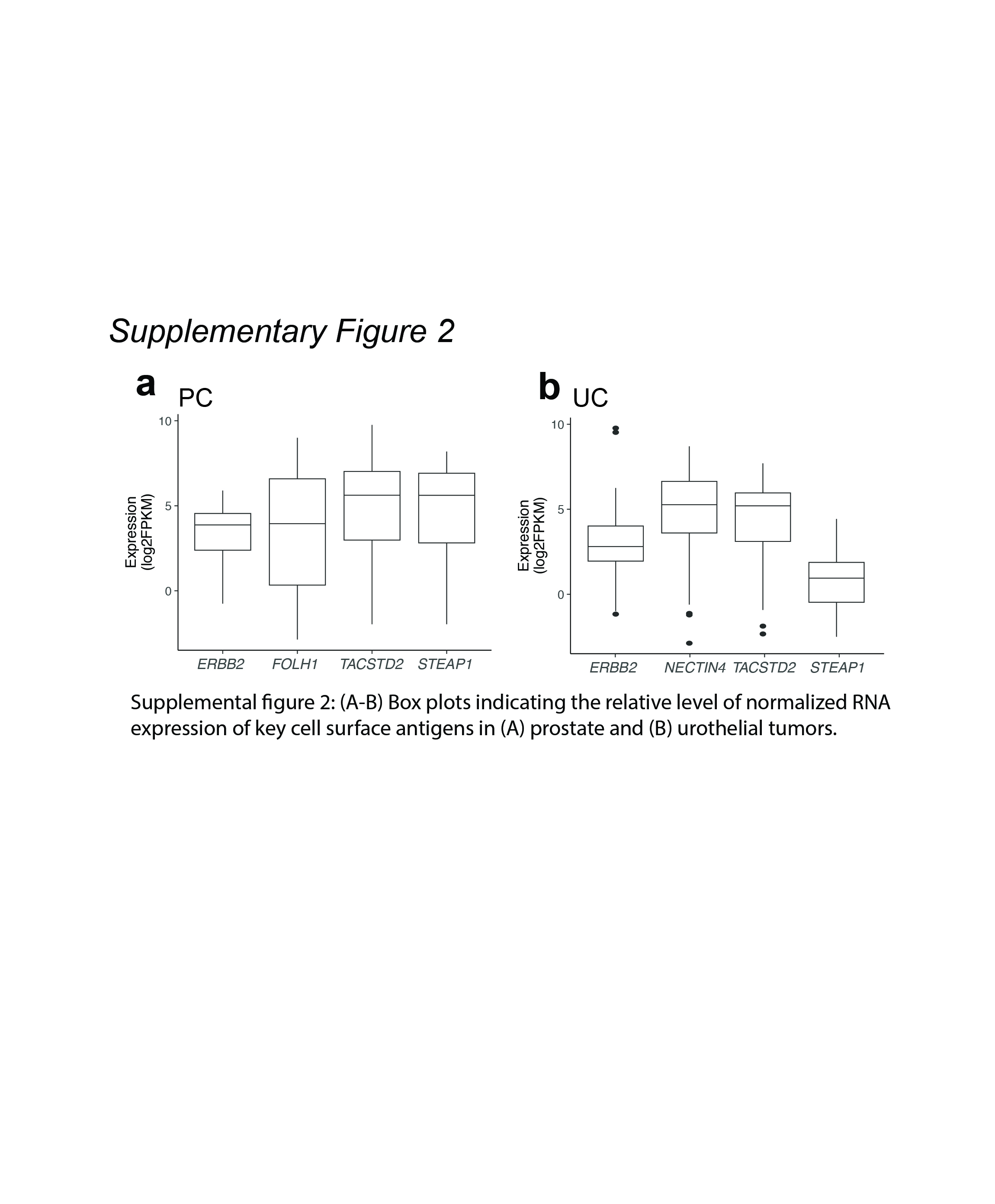

Supplement: Figure S2 — Supplemental figure 2 [file crc-25-0069_figure_s2_suppsf2.jpeg]
